# HPV type concordance in sexual couples determines the effect of condoms on regression of flat penile lesions

**DOI:** 10.1038/sj.bjc.6602524

**Published:** 2005-04-05

**Authors:** M C G Bleeker, J Berkhof, C J A Hogewoning, F J Voorhorst, A J C van den Brule, T M Starink, P J F Snijders, C J L M Meijer

**Affiliations:** 1 Department of Pathology, VU University Medical Center, Amsterdam, the Netherlands; 2 Department of Clinical Epidemiology and Biostatistics, VU University Medical Center, Amsterdam, the Netherlands; 3 Department of Gynaecology and Obstetrics, Albert Schweitzer Hospital, Dordrecht, the Netherlands; 4 Department of Dermatology, VU University Medical Center, Amsterdam, the Netherlands

**Keywords:** condom use, flat penile lesions, HPV type concordance

## Abstract

We earlier demonstrated, in a randomised clinical trial, that the regression time of flat penile lsions in male sexual partners of women with cervical intraepithelial neoplasia (CIN) was shorter in men who used condoms compared to those who did not. To further evaluate this finding, we examined whether the effect of condom use on the regression of flat penile lesions depends on the presence of human papillomavirus (HPV) type concordance in sexual couples, as determined in cervical and penile scrapes by GP5+/6+ PCR testing. A Cox model with time-dependent covariates showed a beneficial effect of condoms on regression of flat penile lesions in concordant couples (hazard ratio 2.63, 95% CI 1.07–6.48) but not in those who were nonconcordant. When both partners harboured different HPV types, no effect of condoms was found (hazard ratio 0.90, 95% CI 0.27–2.96). Delayed regression of flat penile lesions was associated with either stable lesions or with new penile lesions developing at sites surrounding pre-existing lesions suggesting reinfection of the penile epithelium. We conclude that condom use blocks sexual HPV transmission by preventing reinfection and development of new penile lesions in men who are susceptible to the same type as present in the female partner.

Infection with high-risk types of human papillomavirus (HPV) is the major cause of cervical cancer and its precursor lesions (Schiffman *et al*, 1993; Walboomers *et al*, 1999; Bosch *et al*, 2002; Munoz *et al*, 2003). Human papillomavirus has also been linked to cancers of the vulva, vagina, anus, and penis (IARC 1995; Melbye and Frisch, 1998). Genital HPV types are transmitted sexually and the risk of infection has been associated with numerous parameters involving sexual behaviour (Franco *et al*, 1995; Bosch *et al*, 1996; Kjaer *et al*, 2001).

Partner studies have shown that penile lesions are found in more than half of the male partners of women with cervical intraepithelial neoplasia (CIN) (Barrasso *et al*, 1987; Campion *et al*, 1988; Hippelainen M *et al*, 1991; Bleeker *et al*, 2002). Flat lesions are reported as the most prevalent lesion type and their relationship with HPV has been established (Barrasso *et al*, 1987; Campion *et al*, 1988; Hippelainen M *et al*, 1991; Hippelainen MI *et al*, 1993b; Bleeker *et al*, 2002). Since flat penile lesions are only well visible after acetowhitening, their presence remains often unnoticed. This explains why the natural history of penile lesions and HPV infection in men has not been studied extensively and is largely unknown.

We have recently shown that the presence of penile HPV is associated with delayed regression whereas condom use revealed accelerated regression (Bleeker *et al*, 2003). The beneficial effect of condom intervention on regression of penile lesions suggests that a sexual transmittable factor, that is, most likely cervical HPV, influences the clinical course of these lesions. To evaluate this hypothesis, the effect of condom use on regression of flat penile lesions was studied in relation to the presence of HPV type concordance between sexual partners. Since use of condoms and presence of concordance could change during this prospective study, effects of condom use were estimated by a Cox model with time-dependent covariates.

## PATIENTS AND METHODS

### Study population

The study population consisted of men recruited at the colposcopy clinic of the Albert Schweitzer Hospital, Dordrecht, the Netherlands, from January 1995 to June 2002. These men participated in a randomised clinical trial, studying the influence of condom use on regression of CIN and penile lesions in sexual couples. Details of this trial have been described recently (Bleeker *et al*, 2003; Hogewoning *et al*, 2003). Men were screened for penile lesions by penoscopy and a penile scrape was taken for HPV PCR testing. Women were screened by colposcopy and scrapes were taken for cytology and HPV testing. Baseline cervical biopsy specimens were taken for histopathological evaluation. Couples were randomised to either condom use or not using a block randomisation scheme with block size two. Condoms were proposed for at least 3 months and randomisation occurred independent of the presence of HPV, penile lesions, grade of CIN lesion, or surgical treatment of the CIN lesion. Exclusion criterion was regular condom use at baseline. Follow-up examinations were carried out after 3 and 6 months and subsequently every 6 months. In the condom arm, condom use was verified at each visit by asking the frequency of failures to use condoms. Partners were not asked separately for the number of failures and, in case of discrepancies between partners, the couples themselves made a consensus. Also, couples assigned to the noncondom arm were asked during each follow-up visit whether they have used condoms but none of them reported condom use. To obtain information on lifestyle habits, including sexual behaviour, couples were asked to complete a questionnaire. This questionnaire was introduced in 1999 and completed, independently by each partner, in separate rooms. The medical ethics review board of the hospital approved the study protocol (protocol number: CGE/95/238) and the participants signed informed consents.

### Data collection

#### Penoscopic examination

Penoscopic examination and specimen collection were performed as described earlier by us (Bleeker *et al*, 2002). Briefly, using a Cervex brush®, a penile scrape for HPV testing was taken to collect cells from the glans, corona, sulcus, frenulum, and inner part of the foreskin. Subsequently, a colposcope was used to identify flat penile lesions after the application of 3% acetic acid solution. Follow-up penoscopic examinations were performed blinded of data from previous visits. Findings were written on a standardised form and penile lesions were documented by digital photography. Photographs were subsequently reviewed and graded by an experienced dermatologist (TMS), unaware of any clinical data. In case of discrepancies (<5%) between the initial finding and the review result, a consensus diagnoses was made with the gynaecologist who made the photographs and the dermatologist who reviewed the photographs.

#### Human papillomavirus testing

Processing of specimens and testing for HPV was carried out at the Department of Pathology, VU University Medical Center, as described previously (Jacobs *et al*, 1997; Bleeker *et al*, 2002). In short, DNA quality was tested by *β*-globin PCR and testing for HPV was carried out using the HPV GP5+/6+ PCR enzyme immunoassay (HPV PCR-EIA), to identify 14 high-risk (16, 18, 31, 33, 35, 39, 45, 51, 52, 56, 58, 59, 66, and 68) and six low-risk (6, 11, 40, 42, 43, and 44) HPV genotypes with a high-risk and a low-risk HPV probe cocktail, respectively. Additionally, individual typing was performed for HPV 16, 18, 31, 33, 6, and 11, using type-specific probes in an EIA format. Samples, which were inadequate for HPV testing, that is, were negative in both *β*-globin and HPV PCR assays, were excluded from the analysis.

### Regression of flat penile lesions

In 75% of the men with flat penile lesions, more than 5 mm^2^ of the penile epithelium was affected and in 94% of them more than one lesion was present (Bleeker *et al*, 2005). Follow-up of individual flat penile lesions was sometimes difficult, especially when lesions were small and present on multiple sites of the penis. Larger lesions were usually localized on the same site of the penile epithelium and could vary in size. Owing to this diverse clinical presentation, regression was defined as disappearance of all detectable flat lesions, that is, no flat lesions at penoscopy.

### Statistical analysis

Differences in characteristics of the condom and the noncondom groups were assessed by use of *χ*^2^ or Student's *t*-tests (two-tailed, *P*<0.05), where appropriate.

The outcome of interest in this study was regression of flat penile lesions. To study the complex interrelationship between HPV presence, concordance, and condom use on the regression of the penile lesions, we choose an approach in which changes in condom use or presence of female HPV infection during the study could be included. Therefore, to examine whether the effect of condom use on regression of flat penile lesions was related to the presence of HPV type concordance in sexual couples, Cox regression analysis with time-dependent covariates (condom use and presence of female HPV) was performed to compute hazard ratios and 95% confidence intervals (CI). In the modelling process, hazard ratios for the effect of condom use were computed using the noncondom group as the reference group in couples with a comparable HPV status.

The HPV status in men was defined as positive or negative when penile scrapes were ever or never HPV positive during the study, respectively (Bleeker *et al*, 2003). Other determinants assessed were CIN grade of the female partner at baseline histology (either ⩽CIN 1 or ⩾CIN 2) and age of the men when entering the study.

Originally, sexual couples were considered as HPV type concordant when both sexual partners were positive for HPV 16, 18, 31, 33, 6, 11, other high-risk HPVs (not HPV type 16, 18, 31, or 33) and/or other low-risk HPV (not HPV type 6 and 11) during the study. This definition was used because of practical reasons that individual typing was only performed for HPV 16, 18, 31, 33, 6, and 11, whereas other high-risk HPVs and other low-risk HPVs each typed as a group. A more restrictive definition of concordance was subsequently used including only the couples that were concordant for 16, 18, 31, 33, 6, and/or 11 and excluding the couples that were concordant for other high-risk and/or other low-risk HPV types.

For Cox regression analyses with time-dependent covariates, Stata 6.0 was used and for the remaining analyses SPSS version 9.0 software.

## RESULTS

### Study population

A total of 139 men had flat penile lesions at baseline. Six of them used condoms at baseline and were excluded from this study. No follow-up results were available from 14 men, and in one female partner the presence of HPV could not be determined due to inadequate sample quality at baseline. The remaining 118 men formed the study population. At baseline, 61 (51.7%) couples started condom use and 57 (48.3%) did not. The median duration of condom use was 5.3 months (range 2.5–53.7). Failures of condom use were reported by six couples with a median of 1.5 times during the follow-up period (range 1–2). The median follow-up time of the study population was 12.5 months (range 2.5–77.5): 12.9 months (range 3.0–68.5) in the noncondom and 12.3 months (range 2.5–77.5) in the condom group (*P*=0.388).

Baseline characteristics of the study population are presented in Table 1 and no statistically significant differences were found between the condom and the noncondom group. The mean age of men was 38.7 years (range 28.1–57.7 years) and of women 36.2 years (range 22.6–54.9). Representative biopsy specimens were available for 111 women: 17 had no CIN (15.3%), 34 CIN 1 (30.6%), 39 CIN 2 (35.1%), and 21 ⩾CIN 3 (18.9%). At baseline, 85.6% of the women and 68.6% of the men were positive for HPV DNA. Human papillomavirus 16 was found in 59.4 and 55.9% of the positive women and men, respectively, and their prevalences were not statistically different in the condom and the noncondom group (*P*=0.496 in women and *P*=0.775 in men). Women with HPV at baseline (*n*=101) had a median duration of HPV infection of 9.2 months and were not statistically different in the condom and noncondom group (*P*=0.852).

During the study, at least one concordant HPV type was found in 57 couples: 32 couples of the condom and 25 couples of the noncondom group (*P*=0.350). Human papillomavirus type concordance was found for HPV 16 in 33 (58%), HPV 18 in 0 (0%), HPV 31 in two (4%), HPV 33 in two (4%), other high-risk HPV type in eight (14%), HPV 6 in one (2%), HPV 11 in zero (0%), other low-risk HPV type in three (5%), and multiple HPV types in eight (14%) couples, that is, HPV 16 and 31 (*n*=1), HPV 16 and other high-risk type (*n*=4), HPV 16 and 6 (*n*=1), HPV 33 and other low-risk type (*n*=1), and other high-risk and other low-risk HPV (*n*=1). Concordance for HPV 16 was equally prevalent in the condom and the noncondom groups (*P*=0.952).

### Effect of condom use on regression of flat penile lesions in concordant and nonconcordant sexual couples

Using a Cox model with time-dependent covariates, we examined whether the effect of condom use on regression of flat penile lesions was related to the presence of HPV type concordance in sexual partners. This analysis showed that the beneficial effect of condoms on regression of flat penile lesions was present in concordant couples (hazard ratio 2.63, 95% CI 1.07–6.48, *P*=0.035, Table 2) but not in those who were nonconcordant. Also, no effect of condoms was found when both partners were positive but nonconcordant (hazard ratio 0.90, 95% CI 0.27–2.96, *P*=0.861). The estimated hazard ratio for the effect of condom use when comparing the concordant and the nonconcordant HPV-positive couples was 2.63/0.90=2.93, 95% CI 0.66–13.07 (*P*=0.159).

When we restricted the analysis to couples concordant for HPV types 6, 11, 16, 18, 31, and 33, which were individually typed during this study, similar effects of condom use were obtained.

Regression of flat penile lesions was not related to the age of the men (hazard ratio 0.99, 95% CI 0.95–1.03, *P*=0.501) or the histological CIN grade (hazard ratio 1.27, 95% CI 0.71–2.25, *P*=0.418). Inclusion of these determinants into the Cox model did not markedly change the hazard ratios for condom use.

In case of a delayed regression of flat penile lesions, we observed that either the lesions were stable or that new penile lesions developed at sites surrounding pre-existing lesions suggesting reinfection of the penile epithelium. Hence, a diverse clinical course of flat penile lesions was noticed which was present in both the condom and the noncondom arm.

## DISCUSSION

Our data, collected in a randomised clinical trial, earlier demonstrated that the regression time of flat penile lesions was shorter in men who used condoms compared to those who did not (Bleeker *et al*, 2003). We hypothesised that sexually transmittable HPV of the women delayed regression of flat penile lesions in their male partner and that condoms could effectively block HPV transmission. In the present study, we examined whether the effect of condom use on regression of flat penile lesions depended on the presence of HPV concordance between sexual partners. Indeed, using a Cox model in which changes on condom use and presence of HPV concordance during this study were included, a beneficial effect of condoms on regression of penile lesions was found for HPV type concordant couples, but not for those who were not concordant.

The presence of penile lesions associated with a given HPV type indicates that the man is susceptible for this particular HPV type. In men with penile lesions who harboured a similar HPV type as their female partner, we observed a delayed regression when couples did not use condoms compared to those who did. This finding suggests that failure of penile lesions to cure depends on reinfection by cervical HPV from the female partner for which the man is apparently susceptible. This is substantiated by the observation that new penile lesions often developed next to pre-existing ones. The beneficial effect of condom use in HPV concordant couples can be explained by blocking HPV transmission during sexual intercourse, thereby preventing reinfection and development of new penile lesions. In nonconcordant couples, we found no effect of condom use on regression of penile lesions. Apparently, these men are not susceptible for the development of new lesions by the HPV type, which is present in the female partner. Consequently, the clinical implication of our findings would be that condoms sort only effect in heterosexual couples that are positive for the same HPV type and that, in these couples, condom use should be continued as long as the female partner is HPV positive. In addition, condom use could also be advocated in sexual couples of whom the woman is HPV positive and the male partner does not have penile lesions since we do not know whether the male partner is susceptible for the HPV type of the woman and thus can acquire a penile lesion.

So far, studies on the effectiveness of condoms in relation to the prevention of HPV infection and related disorders were mainly focused on women and available data were too inconsistent to provide precise estimates (Manhart and Koutsky, 2002). Only a few studies were performed in men and reported that consistent condom use reduced the risk for genital warts or subclinical HPV infection (Hippelainen M *et al*, 1993a; Wen *et al*, 1999). Of interest is the work of Hippelainen MI *et al* (1994) who found that condom use had a positive effect on the cure rate of genital HPV lesions in male partners of HPV-infected women. However, all studies evaluating condom use were either cross-sectional or observational and not explicitly designed to evaluate condom use prospectively.

In this partner study, however, it should be realised that grouping into concordant and nonconcordant may be subject to some degree of underestimation of concordant sexual couples. Nondetection of HPV in penile scrapes does not necessarily reflect absence of HPV (Bleeker *et al*, 2003). Compared to cervical scrapings, low cell numbers are collected by scraping the penile epithelium and consequently low numbers of infected cells might have been missed during the sampling. Therefore, because of potential false negativity in penile scrapes, the currently defined group of nonconcordant couples may contain some concordant couples as well. This may explain the hazard ratio of 2.40 (95% CI 0.47–12.36) in nonconcordant couples in whom the woman was HPV positive and the man negative. Another issue is that we did not ask the couples to keep a log of their condom use. Therefore, recall bias cannot be completely excluded but it is unlikely that this substantially influenced the data as the number of failures to use condoms was low. Moreover, failures to use condoms would most likely lower the positive effect of condoms on regression of the penile lesions, meaning that the effect of condoms would even be more beneficial when there were no failures of condom use at all.

In conclusion, the beneficial effect of condoms on regression of flat penile lesions was only found when sexual couples were HPV concordant. The effect of condoms is probably the result of blocking transmission of female HPV to the penis, thereby preventing reinfection and induction of a penile lesion to which a man is susceptible. As HPV testing becomes more widely used both for the management of women with HPV-related disorders and in cervical screening programs, accurate information on how to protect against HPV is needed. Our data suggest that condom use can reduce the spread of HPV in a sexually active population. Our findings warrant further studies on this topic.

## Figures and Tables

**Table 1:** Baseline characteristics of the study population

			**Condom−**		**Condom+**	
	***N***	**%**	***N***	**%**	***N***	**%**
*Subjects*	118	*100*	57	*48.3*	61	*51.7*
*Age*						
Men	38.7		38.9		38.5	
Women	36.2		36.1		36.3	
			
*Histological CIN grade in female partner*						
No (representative) biopsy	7		4		3	
No CIN	17	*15.3*	10	*18.9*	7	*12.1*
CIN 1	34	*30.6*	15	*28.3*	19	*32.8*
CIN 2	39	*35.1*	15	*28.3*	24	*41.4*
⩾CIN 3	21	*18.9*	13	*24.5*	8	*13.8*
						
*Cervical HPV*						
Negative	17	*14.4*	11	*19.3*	6	*9.8*
Positive	101	*85.6*	46	*80.7*	55	*90.2*
						
*Penile HPV*						
Inadequate^a^	32		19		13	
Negative	27	*31.4*	12	*31.6*	15	*31.3*
Positive	59	*68.6*	26	*68.4*	33	*68.8*

a Could not be determined because samples were inadequate for HPV testing. HPV=human papillomavirus; CIN=cervical intraepithelial neoplasia.

**Table 2:** Effect of condom use on regression of flat penile lesions

		**Effect of condom use^a^**		
**Cervical HPV**	**Penile HPV**	**Hazard ratio**	**95% CI**	***P***
*Nonconcordant*	*Nonconcordant*			
Negative	Negative	1.02	0.10–10.11	0.986
	Positive	1.04	0.07–15.55	0.978
Positive	Negative	2.40	0.47–12.36	0.294
	Positive	0.90	0.27–2.96	0.862
				
*Concordant*	*Concordant*			
Positive	Positive	**2.63**	**1.07–6.48**	**0.035**

a Cox regression analyses with time-dependent predictors (cervical HPV and condoms) were performed to compute 95% CI of the hazard ratios for the effect of condom use *vs* no use of condoms. Boldface numbers indicate statistical significance. CI=confidence interval; HPV=human papillomavirus.

## References

[bib1] Barrasso R, de Brux J, Croissant O, Orth G (1987) High prevalence of papillomavirus-associated penile intraepithelial neoplasia in sexual partners of women with cervical intraepithelial neoplasia. N Engl J Med 317: 916–923 3041217 10.1056/NEJM198710083171502

[bib2] Bleeker MC, Hogewoning CJ, van den Brule AJ, Voorhorst FJ, Van Andel RE, Risse EK, Starink TM, Meijer CJ (2002) Penile lesions and human papillomavirus in male sexual partners of women with cervical intraepithelial neoplasia. J Am Acad Dermatol 47: 351–357 12196743 10.1067/mjd.2002.122198

[bib3] Bleeker MC, Hogewoning CJ, Voorhorst FJ, van den Brule AJ, Snijders PJ, Starink TM, Berkhof J, Meijer CJ (2003) Condom use promotes regression of human papillomavirus-associated penile lesions in male sexual partners of women with cervical intraepithelial neoplasia. Int J Cancer 107: 804–810 14566831 10.1002/ijc.11473

[bib4] Bleeker MC, Hogewoning CJ, Voorhorst FJ, van den Brule AJ, Snijders PJ, Starink TM, Berkhof J, Meijer CJ (2005) HPV associated flat penile lesions in men of a non-STD hospital population: less frequent and smaller in size than in male sexual partners of women with CIN. Int J Cancer 113: 36–41 15386360 10.1002/ijc.20502

[bib5] Bosch FX, Castellsague X, Munoz N, de Sanjose S, Ghaffari AM, Gonzalez LC, Gili M, Izarzugaza I, Viladiu P, Navarro C, Vergara A, Ascunce N, Guerrero E, Shah KV (1996) Male sexual behavior and human papillomavirus DNA: key risk factors for cervical cancer in Spain. J Natl Cancer Inst 88: 1060–1067 8683637 10.1093/jnci/88.15.1060

[bib6] Bosch FX, Lorincz A, Munoz N, Meijer CJ, Shah KV (2002) The causal relation between human papillomavirus and cervical cancer. J Clin Pathol 55: 244–265 11919208 10.1136/jcp.55.4.244 PMC1769629

[bib7] Campion MJ, McCance DJ, Mitchell HS, Jenkins D, Singer A, Oriel JD (1988) Subclinical penile human papillomavirus infection and dysplasia in consorts of women with cervical neoplasia. Genitourin Med 64: 90–99 2838408 10.1136/sti.64.2.90 PMC1194164

[bib8] Franco EL, Villa LL, Ruiz A, Costa MC (1995) Transmission of cervical human papillomavirus infection by sexual activity: differences between low and high oncogenic risk types. J Infect Dis 172: 756–763 7658069 10.1093/infdis/172.3.756

[bib9] Hippelainen M, Syrjanen S, Hippelainen M, Koskela H, Pulkkinen J, Saarikoski S, Syrjanen K (1993a) Prevalence and risk factors of genital human papillomavirus (HPV) infections in healthy males: a study on Finnish conscripts. Sex Transm Dis 20: 321–328 8108754

[bib10] Hippelainen M, Yliskoski M, Saarikoski S, Syrjanen S, Syrjanen K (1991) Genital human papillomavirus lesions of the male sexual partners: the diagnostic accuracy of peniscopy [see comments]. Genitourin Med 67: 291–296 1655625 10.1136/sti.67.4.291 PMC1194703

[bib11] Hippelainen MI, Hippelainen M, Saarikoski S, Syrjanen K (1994) Clinical course and prognostic factors of human papillomavirus infections in men. Sex Transm Dis 21: 272–279 7817261 10.1097/00007435-199409000-00005

[bib12] Hippelainen MI, Syrjanen S, Hippelainen MJ, Saarikoski S, Syrjanen K (1993b) Diagnosis of genital human papillomavirus (HPV) lesions in the male: correlation of peniscopy, histology and *in situ* hybridisation [see comments]. Genitourin Med 69: 346–351 8244350 10.1136/sti.69.5.346 PMC1195115

[bib13] Hogewoning CJ, Bleeker MC, van den Brule AJ, Voorhorst FJ, Snijders PJ, Berkhof J, Westenend PJ, Meijer CJ (2003) Condom use promotes regression of cervical intraepithelial neoplasia and clearance of human papillomavirus: a randomized clinical trial. Int J Cancer 107: 811–816 14566832 10.1002/ijc.11474

[bib14] IARC Monographs on the Evaluation of Carcinogenic Risks to Humans (1995) Human papillomaviruses vol. 64. Lyon: IARC Scientific publications

[bib15] Jacobs MV, Snijders PJ, van den Brule AJ, Helmerhorst TJ, Meijer CJ, Walboomers JM (1997) A general primer GP5+/GP6+ mediated PCR-enzyme immunoassay method for rapid detection of 14 high-risk and 6 low-risk human papillomavirus genotypes in cervical scrapings. J Clin Microbiol 35: 791–795 9041439 10.1128/jcm.35.3.791-795.1997 PMC229677

[bib16] Kjaer SK, Chackerian B, van den Brule AJ, Svare EI, Paull G, Walbomers JM, Schiller JT, Bock JE, Sherman ME, Lowy DR, Meijer CL (2001) High-risk human papillomavirus is sexually transmitted: evidence from a follow-up study of virgins starting sexual activity (intercourse). Cancer Epidemiol Biomark Prev 10: 101–106 11219765

[bib17] Manhart LE, Koutsky LA (2002) Do condoms prevent genital HPV infection, external genital warts, or cervical neoplasia? A meta-analysis. Sex Transm Dis 29: 725–735 12438912 10.1097/00007435-200211000-00018

[bib18] Melbye M, Frisch M (1998) The role of human papillomaviruses in anogenital cancers. Semin Cancer Biol 8: 307–313 9870037 10.1006/scbi.1998.0081

[bib19] Munoz N, Bosch FX, de Sanjose S, Herrero R, Castellsague X, Shah KV, Snijders PJ, Meijer CJ (2003) Epidemiologic classification of human papillomavirus types associated with cervical cancer. N Engl J Med 348: 518–527 12571259 10.1056/NEJMoa021641

[bib20] Schiffman MH, Bauer HM, Hoover RN, Glass AG, Cadell DM, Rush BB, Scott DR, Sherman ME, Kurman RJ, Wacholder S (1993) Epidemiologic evidence showing that human papillomavirus infection causes most cervical intraepithelial neoplasia. J Natl Cancer Inst 85: 958–964 8388478 10.1093/jnci/85.12.958

[bib21] Walboomers JM, Jacobs MV, Manos MM, Bosch FX, Kummer JA, Shah KV, Snijders PJ, Peto J, Meijer CJ, Munoz N (1999) Human papillomavirus is a necessary cause of invasive cervical cancer worldwide [see comments]. J Pathol 189: 12–19 10451482 10.1002/(SICI)1096-9896(199909)189:1<12::AID-PATH431>3.0.CO;2-F

[bib22] Wen LM, Estcourt CS, Simpson JM, Mindel A (1999) Risk factors for the acquisition of genital warts: are condoms protective? Sex Transm Infect 75: 312–316 10616354 10.1136/sti.75.5.312 PMC1758246

